# Prevalence, Antimicrobial Susceptibility Pattern and Associated Factors of* Staphylococcus Aureus* Among Camel's Raw Milk in Babile District, Oromia, Ethiopia

**DOI:** 10.1002/vms3.70438

**Published:** 2025-06-01

**Authors:** Ahmednur Abdi, Siraj Hussen, Mohammed Ahmed

**Affiliations:** ^1^ Department of Medical Laboratory Science College of Medicine and Health Science Dire Dawa University Dire Dawa Ethiopia; ^2^ Department of Medical Laboratory Science College of Medicine and Health Science Hawassa University Hawassa Ethiopia; ^3^ School of Medical Laboratory Science College of Health and Medical Science Haramaya University Harar Ethiopia

**Keywords:** Antibiotic susceptibility profile, Babile district, Prevalence, Raw Camel Milk, *Staphylococcus aureus*

## Abstract

**Background:**

*Staphylococcus aureus* (*S. aureus*) is a versatile pathogen that causes diseases. Raw milk is an ideal, rich medium that helps and supports the growth of microorganisms and is highly susceptible to *S. aureus* immediately after milking. As camel milk was usually consumed in its raw state in pastoralist areas, the contamination and intoxication of raw milk due to pathogenic *S. aureus* were a public health problem.

**Objective:**

The study aimed to determine the prevalence, antibiotic resistance pattern, and associated factors of *S. aureus* in raw camel milk in Babile District, Oromia Region, Eastern Ethiopia.

**Method:**

A community‐based cross‐sectional study was conducted in the Babille district among 350 raw camel milk. Participants were selected using a multi‐stage sampling technique. Data was collected using a pretested structured questionnaire, and 25 mL of raw camel milk was collected in sterile screw‐capped bottles. *S. aureus* was identified through culture, Gram stain, and biochemical tests. A bivariate and multivariable logistic regression was done. *p*‐value ≤ 0.05 was considered statistically significant.

**Result:**

In this study, the overall prevalence of *S. aureus* in raw camel milk was 14% (95% CI: 10, 18). Parity [AOR = 3.52, 95% CI: (1.207, 10.277), *p* = 0.021], drainage condition of the milking place [AOR = 4.62, 95% CI: (1.853, 11.557), *p* = 0.001], not hand washing before milking [AOR = 3.94, 95% CI: (1.599, 9.716), *p* = 0.003] and the type of containers used for selling milk [AOR = 8.40, 95% CI: (1.258, 26.068), *p* = 0.028] were significant predictors of *S. aureus*. A high level of resistance was recorded against tetracycline (81.6%), penicillin G (81.6%), and amoxicillin (69.4%).

**Conclusion:**

Overall, the prevalence of *S. aureus* isolated from raw camel milk of the Babile district was high. Multi‐drug resistance among the isolates was also high. Therefore, washing hands and milking containers before milking and using stainless steel containers instead of plastic containers could be applied. Raw camel milk intended for human consumption should be properly transported, stored and subjected to heat treatment. Increasing awareness and creation of the hygienic practice of camel milk handling is paramount. Antibiotics should only be used to treat sick animals based on the diagnosis of the diseases (Supplementary Information).

## Graphical abstract

This graphical abstract text can be better represented through illustrations for milk samples, bacteria, risk factors (e.g., handwashing, containers), and antibiotic resistance patterns for clearer understanding and to increase engagement.

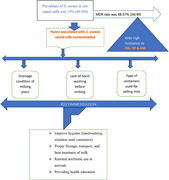

**Summary**

- Prevalence: 14% (95% CI: 10, 18) of *S. aureus*‐positive samples were contaminated.
- The factors associated were:
  ○ Poor drainage in milking areas (AOR = 4.62, *p* = 0.001).
  ○ Lack of handwashing before milking (AOR = 3.94, *p* = 0.003).
  ○ Use of plastic containers for milk storage (AOR = 8.40, *p* = 0.028).
- Antibiotic Resistance Shows High Resistance to Tetracycline (81.6%), Penicillin G (81.6%), and Amoxicillin (69.4%).

## 1 Introduction

Food‐borne diseases are a serious public health threat, which leads to significant losses in productivity and high medical costs (Garcia et al. 2020). Raw milk and raw milk products concerning *staphylococcal* poisoning were of great concern around the world (Sema et al. 2019). According to the World Health Organization (WHO), bacteria were incriminated in two‐thirds of dairy food borne outbreaks, registered globally (Enquebaher 2016). *Staphylococcal* food poisoning (SFP) was estimated to cause 420,000 deaths every year and generate health costs and economic losses in the range of 110 billion dollars worldwide (Danai et al. 2020). Camel milk is a vital source of food, nutrition security, and household income, as well as a significant cultural value for pastoral populations in Eastern African countries including Somalia, Sudan, Ethiopia and Kenya (Guliye et al. 2007).

Milk, being an essential source of food in the human diet, is an important carrier of both helpful and harmful microbes. Several pathogens, including *Brucella spp., Campylobacter spp*., Shiga toxin‐producing *Escherichia coli, Listeria monocytogenes, Mycobacterium spp., Salmonella spp*., and bacterial toxins, have been causally associated with milk‐borne illnesses (Dhanashekar et al. 2012). Among these bacteria, *Staphylococcus aureus (S. aureus)* is most commonly responsible for SFP outbreaks, and the ingestion of less than 1.0 µg enterotoxin causes SFP (Abdurabbah et al. 2018). Furthermore, zoonotic bacteria have developed significant antibiotic resistance. Antibiotics are utilised extensively in food animal production in underdeveloped nations to improve animal health and growth, even if their use in camels is not that much studied (Woldearegay et al. 2015, Gemeda et al. 2020). This approach may give some economic benefits to producers and consumers in general. Nonetheless, a major risk in this practice is related to continually exposing these animals to modest dosages of antibiotics that contribute considerably to antimicrobial resistance (Van et al. 2020).

*S. aureus* is a ubiquitous pathogen that causes invasive and life‐threatening infections, ranging in severity from slight skin infections to more severe diseases such as pneumonia, endocarditis, and septicaemia (Yakubu et al. 2020, Teshome et al. 2016)*. S. aureus* is a Gram‐positive, catalase‐positive, facultative anaerobic, and usually oxidase‐negative bacterium which belongs to the family of *Micrococcaceae* and the genus *Staphylococcus* (Aqib et al. 2018). The natural ecological niches of *S. aureus* are the nasal cavity and the skin of warm‐blooded animals. The mucous membranes, skin, udders, and teats of milking animals are the most important reservoirs of this contaminant (Teshome et al. 2016).

Methicillin‐resistant *S. aureus* (MRSA) and biofilm‐producing *S. aureus* are emerging common strains of *S. aureus* being isolated from dairy products (Aqib et al. 2019). It produces a variety of toxins and invasive enzymes such as haemolysins, staphylococcal enterotoxins (SE), toxic shock syndrome toxin‐1 (TSST‐1), leukocidin, plasma coagulase and deoxyribonuclease (Jingsha et al. 2019). There are several types of *staphylococcal enterotoxins* (SE), and SE is responsible for dairy food poisoning, and to date, more than 21 different SEs and SE‐like super‐antigens have been identified (Betelihem and Shimels 2017). Among various factors associated with this pathogen are unhygienic milking procedures, poor handling practices of milk, improper preventive techniques, lack of germicidal teat dipping, and improper storage and transport of milk (Ayoub et al. 2020). In addition, dairy animals suffer from *S. aureus*‐induced mastitis is also the other source of milk contamination (Remaz et al. 2017). Furthermore, in camels, anatomically, the anus is positioned above the udders, making faecal contamination during milking a common occurrence, even in the most hygienic operations (Dairy safe, 2019).

*S. aureus* has been associated with many diseases of humans and animals, and the pathogenicity of this bacterium is mostly due to a combination of genetic factors mediating invasiveness, virulence, ability to produce different enzymes, antibiotic resistance, ability to evade the immune system of the host, and possessing mechanisms that damage the host's tissue and facilitate colonisation (Emmanuella et al. 2019, Arumugam et al. 2017, Edalati et al. 2019). Raw milk is an ideal rich medium that supports the growth of microorganisms and is an important source of *staphylococcal* food poisoning that causes gastroenteritis (Jingsha et al. 2019). Milk is highly susceptible to *S. aureus* immediately after milking when it is almost at body temperature (Tawfik et al. 2019).

There are many ways by which the pathogen can enter into dairy food destined for human consumption, especially in the raw state (Ana et al. 2020). Most commonly, microbial contamination of milk can occur from three main sources: from within the udder, from the exterior of the udder, and from the surface of milk handling and storage equipment (Ewa et al. 2016). Approximately 20–30% of human populations are consistent carriers of this bacterium, while 60% are the transient carriers of *S. aureus*. Therefore, insufficient pasteurisation during preparation, processing, and distribution by the carriers of *S. aureus* is the common factor for the outbreaks of staphylococcal dairy food poisoning (Fakhri et al. 2019).

In developing countries like Ethiopia, there is high consumption of raw milk with poor hygienic practices (Carruth et al. 2017). Also, misuse of antibiotics was common, which may result in the development of multi‐drug resistant (MDR) *S. aureus* isolates, and *S. aureus* milk intoxication was one of the leading public health problems in this regard (Yenealem 2020). In Ethiopia, the data regarding the prevalence of *S. aureus* in raw camel milk was scarce. Some authors have reported the prevalence of *S. aureus* in a limited area of the country, with varied prevalences ranging from 4.2% (n = 174) at Jigjiga town (Husein et al. 2013) to 88% (n = 35) at Dubti town in the Afar region (Wasie et al. 2015), which needs national surveillance to get the general epidemiology of *S. aureus in* raw camel milk. Generally, there are few studies on the prevalence, associated factors, and antimicrobial susceptibility of *S. aureus* in raw camel milk in Ethiopia, and more extensive investigations may provide more information. Continuous monitoring of the prevalence and profile of antimicrobial susceptibility could help to control infections more effectively while reducing the emergence of antibiotic‐resistant microbes.

Therefore, this study was aimed at determining the prevalence, antimicrobial susceptibility pattern, and associated factors of *S. aureus* in raw camel milk in the Babille district Oromia region of Eastern Ethiopia.

## 2 Methods and Materials

### 2.1 Study Setting, Design and Period

The study was conducted at Babile District, which is located in the East Hararghe zone, Oromia region, Eastern Ethiopia, from May 2021 to July 2021. Babile District (BD) is one of the districts in the East Hararghe zone located in the eastern corner of Oromia Regional State, bordered by Fedis, Gursum, Harari and Somali National Regional State. The district has a total area of 3169.06 km^2^ and an estimated total population of 94,650 people, of whom 56,198 are male and 48,452 are female (CSA 2014). Babille district has 20 rural kebeles and two urban administrations. Babile district has a huge potential in dairy production and livestock husbandry, which was dominated by 56,355 cattle, 122,160 sheep, 23,020 goats and 10,317 camels (Amentie et al. 2016, District 2018). According to evidence obtained from the Babille district, average milk production per year was about 3,470,776 litres, and about 75% was used for the market as income generation in the district (BDLDHA 2018). A community‐based cross‐sectional study was conducted among raw camel milk in the Babille district. The unavailability of raw milk during sample collection was excluded from the study.

### 2.2 Sample Size and Sampling Technique

The sample size (**n** = **350**) was calculated using a single population proportion formula with the following assumptions: confidence level of 95%, margin of error of 5%, prevalence of *S. aureus* in raw camel's milk in Jigjiga 11.45% (Serda et al. 2018), design effect 2 and 10% non‐respondent rate.

**FIGURE 1:**
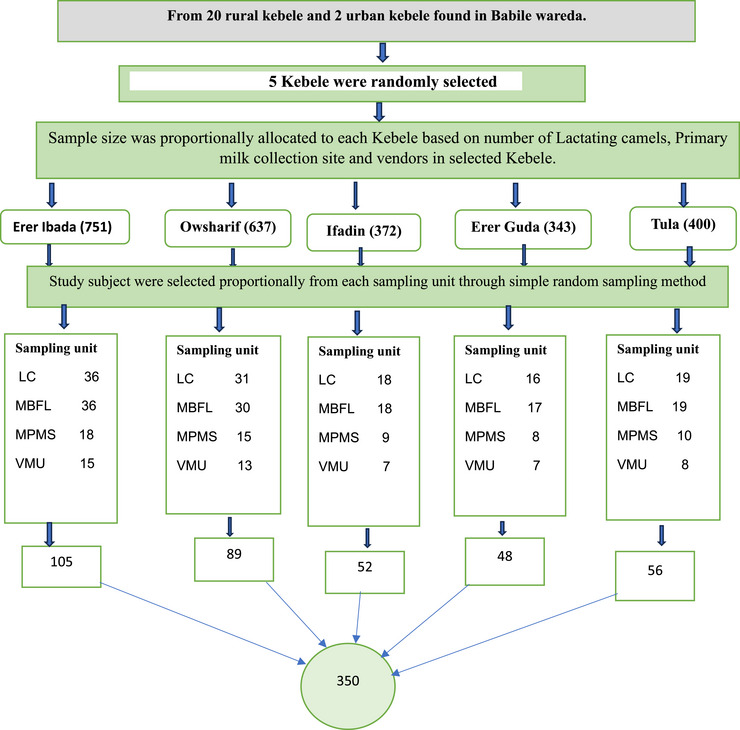
Diagram showing sampling technique from Babille district, Oromia Region Eastern Ethiopia, 2021. LC, lactating camel; MPMS, milking bucket at farm level; MPMS, Milk utensil at primary milk collection site; VMU, vendors milk utensil.

Multi‐stage sampling techniques were used to select kebeles, dairy householders, primary milk collectors, vendors, and milk utensils. From 20 rural kebele and 2 urban administrative, of which 5 kebele were selected using the lottery method. Accordingly, 350 respondents and subsequent 350 camels’ raw milk samples owned by these respondents were aseptically collected from the udder, milking bucket at farm level, milk container from primary milk collectors, and vendor's milk container using simple random sampling. After proportional allocation was made to take an appropriate sample from woredas, we took 105 from Erer ibada, 89 from Owsharif, 52 from Ifadin, 48 from Erer guda and 56 from Tula kabale according to the following diagram (Figure 1).

### 2.3 Data Collection Methods

Data collectors were trained and informed to collect data by using the structured questionnaire. After informed, voluntary, written, and signed consent was obtained, socio‐demographic characteristics and information related to milk handling practices such as awareness of milk contamination, transportation condition, storage condition, drainage condition of milking environment, body condition scour of lactating camels, washing of milking equipment and milk storage, hand washing before milking, type of the milking equipment, type of water used, filtration of milk after milking, stage of lactation, parity of the camel, age of lactating camel and heard size was collected by trained medical laboratory profession through face‐to‐face interview using structured questionnaires. While administering questionnaires, direct observation of the cleanliness of body condition scour and drainage condition of the milking place was also noted.

### 2.4 Sample Collection Procedure and Laboratory Analysis

Twenty‐five (Ewa et al. 2016) mL of fresh milk samples were collected into sterile screw‐capped bottles by trained laboratory technologists. Samples from the udder were collected directly from the udder of randomly selected lactating camels on the farm. The surface of the teat end was cleaned by wiping it with clean cotton dipped in 70% alcohol before sampling, and the first jets of milk were discharged to reduce the contamination of the teat canal (Venugopal et al. 2019).

The bottles were labelled with permanent markers before sampling. All samples were placed in separate sterile plastic bags to prevent spillage and cross‐contamination, and sample‐containing bottles were transported in an icebox to the Microbiology laboratory, College of Health and Medical Sciences, Haramaya University within 2 h of collection. Upon arrival, the samples were cultured immediately or stored in a refrigerator at 4°C for a maximum of 24 h until inoculated onto a standard bacteriological media (Yenealem 2020).

The bacteriological medium was prepared according to the manufacturer's recommendations, and milk samples were subjected to bacterial culture and identification according to the procedures described by Quinn (Quinn et al. 2004). The presence of *Staphylococcus* was confirmed based on colony morphology, Gram's staining, catalase tests and DNase tests. Briefly, a loop full of milk samples was inoculated onto blood agar base enriched with 5% sheep blood (S2) and incubated aerobically at 37^∘^C for 24–48 hrs. Suspected colonies were sub‐cultured onto mannitol salt agar (S1) and incubated aerobically at 37°C for 24–48 hrs. The colonies of staphylococci that produced a yellow pigment on mannitol salt agar media were subjected to DNase tests. Finally, *Staphylococcus aureus* was identified as DNase positive (Quinn et al. 2004). After identification, all isolates of *S. aureus* were preserved at ‐20°C in 30% glycerol.

### 2.5 Antimicrobial Susceptibility Tests

Antibiotic susceptibility testing was done using the Kirby‐Bauer disc diffusion method on Mueller‐Hinton agar, according to the criteria set by the Clinical and Laboratory Standards Institute (CLSI 27^th^ edition) (Clinical and Laboratory Standards Institute (CLSI) 2017). The following antimicrobial discs were used: amoxicillin (25 µg), penicillin G (10 units), cefoxitin (30 µg), gentamicin (10 µg), ciprofloxacin (5 µg), trimethoprim‐sulfamethoxazole (25 µg), erythromycin (15 µg), chloramphenicol (30 µg), oxacillin (1 µg) and tetracycline (30 µg) (Teshome et al. 2016). These antibiotics were selected because of their local availability, and antibiotics of veterinary and human health relevance were also considered.

Fresh overnight cultures were prepared and used for antibiotic sensitivity tests. About 3–5 morphologically identical colonies of *S. aureus* from pure cultures were collected with an inoculating loop and transferred into a tube containing 5 mL of nutrient broth, and the suspension became adjusted to the density of 0.5 McFarland standards, which yielded a uniform suspension. After incubating the plates at 37°C for 18–24 h aerobically, diameters of the zone of bacterial growth inhibition around the discs were measured to the nearest millimetre, and the susceptibility or resistance to the agent in each disc was determined (S3). *S. aureus* susceptibility was classified as sensitive, intermediate, or resistant according to the standardised table of CLSI (2017) (Clinical and Laboratory Standards Institute (CLSI) 2017).

### 2.6 Data Management and Quality Control

The questionnaire was prepared in English, translated to Afan Oromo, and then translated back to English to check for consistency. Proper training was given to data collectors. Before starting the actual study, the questionnaire was pre‐tested in non‐selected kebele (Gamachu and Darera Arba kebele), and feedback was presented to the data collectors, and the questionnaire was amended accordingly. The principal investigator monitored the data collection process to ensure the completeness and reliability of the collected information throughout the data collection process.

For laboratory analysis, pre‐analytical, analytical, and post‐analytical stages of quality assurance were applied, and standard operating procedures (SOPs) of the microbiology laboratory were strictly followed. New batches of stain and reagent were checked for the correct staining reaction using a smear containing known Gram‐positive and Gram‐negative *S. aureus* as a control. Preparation and performance evaluation of culture media were improved by strictly following standard operating procedures and the manufacturer's instructions. All culture plates and antibiotic discs were stored at the recommended refrigeration temperature (2–8°C). Sterility of culture media was assessed by incubating 3–5% of a batch of prepared culture media at 35–37° for 24 h and checking for any growth. The reference strain of *S. aureus* ATCC 25923 was used for quality control of the antimicrobial susceptibility test. Quality control of culture media was checked by inoculating quarter plates of the medium with a control organism and incubating aerobically at 35–37°C for 24 h. The results of each control species and isolated *S. aureus* were recorded.

### 2.7 Data Processing and Analysis

Data was checked for completeness, cleaned, coded, and entered using EPI Data version 3.1 and exported to the statistical package for social scence (SPSS) version 25 for analysis. All variables were initially tested for association with *S. aureus* in raw camel milk by using the binary logistic regression. Then the variables, which showed *p* < 0.25 by binary logistic regression, were selected for multivariate analysis to check for possible associated factors of *S. aureus* by controlling potential confounding factors. An odds ratio with a 95% confidence interval (CI) was used to measure the strength of the association. In all cases, a P‐value <0.05 was considered to be statistically significant.

## 3 Results

### 3.1 Sociodemographic Characteristic of Respondents in Babile District

In this study, 350 respondents were enrolled with a 100% response rate. Of these, 120 were camel householders, 120 were milking personnel, 60 were primary milk collectors and 50 were vendors. The mean age of the respondents was 35.14 with (±SD) 7.99 years. Of the total respondents, 68.6% were females, 92.7% were rural residents and 77.7% of respondents had no formal education (Table 1).

**TABLE 1:** Sociodemographic characteristic of respondents in Babile district, Oromia, Eastern Ethiopia, May to July, 2021(N= 350)

Variables	Category	Number	Percent (%)
Age	18–20	17	4.9%
	21–30	81	23.1%
	31–40	163	46.6%
	41–50	89	25.4%
Sex	Male	110	31.4%
	Female	240	68.6%
Residence	Urban	27	7.7%
	Rural	323	92.3%
Level of education	No formal education	272	77.7%
	Primary	49	14%
	Secondary	26	7.4%
	College and above	3	0.9%

### 3.2 Prevalence of *S. aureus* in Raw Camel Milk of Babile District

The overall prevalence of *S. aureus* isolated from raw camel milk in the study area was 14% (95% CI; 10–18). Out of the total sample size, 26.6% (13/49), 30.6% (15/49), 22.4% (11/49) and 20.4% (10/49) were isolated samples from the udder, milking container, primary milk collection centre, and vendor's milk container, respectively. The prevalence within the sources of sampling points varied from 10.8 % to 20 %: 10.8 %, 12.6%, 18.3% and 20 % were isolated from udder, milking container, primary milk collection centre and vendor's milk container, respectively. There was variation in the prevalence of *S. aureus* between kebeles.

Among the five‐selected kebeles, the higher prevalence of *S. aureus* was isolated from the Erer Gudda kebele at 20.8% (10/48), followed by Ifadin at 15.4% (8/52), while the lowest was isolated from the Owsharif kebele at 11.2% (10/89).

### 3.3 Factors Associated With Prevalence of *S.aureus* in Raw Camel Milk

#### 3.3.1 Factors Related to Milk Handling Practice by Milking Personnel and Lactating Camels

In this study, 61.7% (148/240) of householders had kept a small number of dairy camels (herd size), and 42.1% were lactating camels that gave few births. A large proportion of milking personnel (70.8%) used plastic containers for milking; most of the milking personnel (94.2%) did not wash the udder before milking, and 93.7% did not use antiseptic during milking (Table 2).

**TABLE 2:** Factors related to milk handling practice by milking personnel and lactating camels in Babile district, Oromia region, Ethiopia, May to July, 2021(n= 240).

Variables	Category	Number	Percent%
Body condition scour of lactating camels	Good	116	48.3%
	Modern	79	32.9%
	Poor	45	18.8%
Number of dairy camels (herd size)	Small (< 5)	148	61.7%
	Medium (5–10)	72	30%
	Large (> 10)	20	8.3%
Number of birth lactating camels gave (Parity).	Few (1–2 calves)	101	42.1%
	Moderate (3–4 calves)	86	35.8%
	Many (> 4 calves)	53	22.1%
Stage of lactation in month	Early (1–2)	85	35.4%
	Medium (3–9)	79	32.9%
	Late (10–18)	76	31.7%
Age of lactating camels	Young (≥ 5–7)	91	37%
	Adult (> 8 ‐≥ 11)	105	43.8%
	Old (> 11)	44	18.3%
Drainage condition	Good	159	66.3%
	Poor	81	33.7%
Type of utensils used	Plastic containers	170	70.8%
	Stainless steel containers	11	4.6%
	Traditional container	59	24.6%
Cleaning of utensils before milking	Yes	231	96.3%
	No	9	3.7%
Cleaning condition	Cold water and Scrub with sand	188	78.3%
	Soap and cold water	22	9.2%
	Soap and hot water	30	12.5%
Smoking of milking equipment	Yes	235	97.9%
	No	5	2.1%
Hand washing before milking	Yes	202	84.2%
	No	38	15.8%
Ways to wash hands before milking	With cold water	155	75.2%
	Soap and cold water	28	13.6%
	Soap and hot water	23	11.2%
Udder washing before milking	Yes	14	5.8%
	No	226	94.2
Udder or teats dried after washing	Yes	11	78.6%
	No	3	21.4%
Use of separate towels	Yes	3	6.3%
	No	11	93.7%
Antiseptic use during milking	Yes	15	21.4%
	No	225	78.6%
Hair of milkers	Covered	213	88.7%
	Not covered	27	11.3%
Milk filtering	Yes	27	11.2%
	No	213	88.8%

### 3.4 Factors Related to Milk Collectors at the Primary Collection Centre, Main Source of Water and Awareness of Risks Associated With Raw Milk Consumption

The present study result showed that from 60 primary milk collection centres, 50% of primary milk collectors used narrow necked‐plastic vessels to collect milk at the primary milk collection centre, and a large proportion of primary milk collectors (41.6%) kept milk at the collection centre for 2–6 h before it was transported to the market. Of 350 respondents, 80% (280/350) used tap water as a main source of water. Moreover, 78.3% (274/350) and 38.3% (134/350) of respondents consumed raw milk and knew of GIT disturbances associated with drinking raw camel milk, respectively (Table 3).

**TABLE 3:** Factors related to milk collectors at the primary collection centre, main source of water, and awareness of risks associated with raw milk consumption in the Babile district, Oromia, Ethiopia, May to July 2021

Variables	Category	Number	Percent (%)
Types of containers used to collect milk	Wide necked‐plastic vessels	20	33.3%
	Narrow necked plastic vessels	30	50%
	Traditional container	10	16.7%
Chilling/cooling machine	Yes	0	0
	No	60	100%
Length of time milk stayed before transportation	< 2 h	13	21.7%
	2 –6 h	25	41.6%
	6 –12 h	22	36.7%
Transportation of milk to the market	Using car	23	38.3%
	Using donkey	28	46.7%
	Motorcycle	9	15%
Main source of water used for cleaning	Tap water	280	80%
	River **/**Open well	70	20%
Habit of milk consumption	Raw milk	274	78.3%
	Boiled milk	76	21.7%
Knowing any GIT disturbance	Yes	134	38.3%
	No	216	61.7%

### 3.5 Factors Related to Vendors' Milk Handling Practice

In this study, 38% (19/50) and 46% (23/50) of vendors sold raw milk and boiled milk respectively. Among 50 vendors, 38% (19/50) and 14% (7/50) used wide‐necked aluminium vessels and used plastic water bottles to sell the milk, respectively. In addition, 50% (25/50) of vendors cleaned their milk containers both before and after the delivery of milk, and 62% (31/50) stored the milk in plastic containers without a cold chain (Table 4).

**TABLE 4:** Factors related to vendors' milk handling practice in Babile district, Oromia region Ethiopia, May to July 2021(n= 50).

Variables	Category	Number	Percent (%)
Type of milk selling	Raw milk	19	38%
	Boiled milk	23	46%
	Both raw and boiled milk	8	16%
Type of containers used for selling milk	Wide‐necked aluminium vessels	19	38%
	Wide‐necked plastic vessels	11	22%
	Narrow necked plastic containers	13	26%
	Used plastic water bottles	7	14%
Routine cleaning of the milk containers.	Cleaning before putting in milk	19	38%
	Cleaning after delivery of milk	6	12%
	Before putting in milk and after delivery of milk	25	50%
Milk storage	In plastic containers	31	62%
	In traditional containers	3	6%
	In stainless steel containers	16	32%
Cooling of milk	Yes	19	38%
	No	31	62%

### 3.6 Bivariate and Multivariable Analysis of Factors Associated With Prevalence of *S. aureus* in Camel's Raw Milk

In bivariate analysis, educational status, body condition scour, number of dairy camels, parity, age of lactating camel, drainage condition of the milking place, how to clean milking utensils, hand washing before milking, hair of milkers, type of milk selling, type of containers used for selling milk, cleaning of milk containers, cooling of milk, types of containers used to deliver milk to the collection centres, the main source of water, the habit of milk consumption, and knowing any GIT disturbance symptoms associated with consumption of raw milk were factors associated with the prevalence of *S. aureus* in a camel's raw milk in binary logistic regression.

However, in multi‐variable analysis, parity [AOR = 3.52, 95% CI: (1.207, 10.277)], drainage condition of milking place [AOR = 4.63, 95% CI: (1.853–11.557)], hand washing before milking [AOR = 3.94, 95% CI: (1.599, 9.716)] and type of containers used for selling milk [AOR = 8.40, 95% CI: (1.258, 26.068)] were significant predictors of the *S. aureus* in raw camel milk (Table 5).

**TABLE 5:** Bivariate and multivariate analysis of factors associated with prevalence of *S. aureus* in camels’ raw milk of Babile district, Oromia Ethiopia, May 2021 to July, 2021(N= 350).

		Prevalence of *S. aureus* in raw camel milks of Babile district		Bivariate analysis		Multivariable analysis	
Variables	Category	Growth n (%)	No Growth n (%)	COR (95% CI)	*p*‐ value	AOR (95% CI)	*p*‐ value
Educational status	No formal Education No formal education	44 (16.2)	228 (83.8)	2.61 (0.598, 11.355)	**0.202** ^*^	1.33 (0.026, 4.315)	0.400
	Primary	3 (6.1)	46 (93.9)	1.88 (0.138, 5.606)	0.893	1.19 (0.050, 12.574)	0.869
	Secondary and college	2 (6.89)	27 (93.103)	1		1	
Body condition scour	Good	9 (7.8)	107 (92.2)	1		1	
	Medium	7 (8.9)	72 (91.1)	1.16 (0.412, 3.244)	0.783	0.65 (0.194, 2.138)	0.473
	Poor	12 (26.7)	33 (73.3)	4.32 (1.675, 11.160)	**0.002** ^*^	1.72 (0.527, 5.615)	0.369
Number of dairy camels (herd size)	Small (< 5)	15 (10.1)	133(89.9)	1		1	
	Medium (5–10)	9 (12.5)	63 (87.5)	1.27 (0.526, 3.051)	0.598	1.02 (0.342, 3.061)	0.966
	Large (>10)	4 (20)	16 (80)	2.21 (0.655, 7.499)	**0.201** ^*^	1.77 (0.137, 4.363)	0.772
Number of birth lactating camels give (parity)	Few (1–2)	7 (6.9)	94 (93.1)	1		1	
	Moderate (3–4)	8 (9.3)	78 (90.7)	1.38 (0.478, 3.967)	0.533	1.11 (0.360, 3.433)	0.854
	Many (> 4)	13 (24.5)	40 (75.15	4.36 (1.621, 11.752)	0.004*	3.52 (1.207, 10.277)	**0.021** ^**^
Age of lactating camels	Young (≥ 5 ≥ 7)	5 (5.5)	86 (94.5)	1		1	
	Adult (≥ 8 ‐≥ 11)	11 (10.5)	94 (89.5)	2.01 (0.672, 6.028)	**0.211** ^*^	3.97 (0.896, 17.622)	0.069
	Old (> 11)	12 (27.3)	32 (72.7)	6.45 (2.106, 19.757)	**0.001** ^*^	3.19 (0.760, 13.419)	0.113
Drainage condition of milking place	Good	9 (5.7)	150(94.3)	1		1	
	Poor	19(23.5)	62 (76.5)	5.11 (2.191, 11.908)	**0.000** ^*^	4.63 (1.853, 11.557)	**0.001** ^**^
How to clean milking utensils	Cold water and Scrub with sand	25 (13.3)	163 (86.7)	4.45 (0.580, 34.119)	**0.151** ^*^	4.43 (0.314, 62.457)	0.136
	Soap and cold water	2 (9.1)	20 (90.9)	2.90 (0.246, 34.187)	0.398	5.31 (0.527, 47.784)	0.136
	Soap and hot water	1 (3.3)	29 (96.7)	1		1	
Washing hand before milking	Yes	15 (7.4)	187(92.6)	1		1	
	No	13 (34.2)	25 (65.8)	6.48 (2.765, 15.197)	**0.000** ^*^	3.94 (1.599, 9.716)	**0.003** ^**^
Hair of milker.	Covered	19 (8.9)	194 (91.1)	1		1	
	Not covered	9 (33.3)	18 (66.7)	5.11 (2.017, 12.921)	**0.001** ^*^	1.87 (0.511, 6.879)	0.344
Type of milk selling	Boiled milk	2 (8.7)	21 (91.3)	1		1	
	Raw milk	6 (31.6)	13 (68.4)	4.85 (0.848, 27.704)	**0.076** ^*^	7.21 (0.956, 54.389)	0.055
	Both Raw and boiled milk	2 (25)	6 (75)	3.50 (0.404, 30.342)	0.256	4. 95 (0.399, 61.438)	0.213
Type of containers used for selling milk.	Wide‐necked aluminium vessels	2 (9.5)	19 (90.5)	1		1	
	Wide‐necked plastic vessels	1 (12.5)	7 (87.5)	1.36 (0.106, 17.417)	0.815	1.70 (0.056, 8.8222)	0.783
	Narrow necked plastic containers	1 (14.3)	6 (85.7)	1.58 (0.121, 20.686)	0.726	1.64 (0.051, 7.965)	0.726
	Used plastic water bottles	6 (42.9)	8 (57.1)	7.13 (1.177, 23.144)	**0.033** ^*^	8.40 (1.258, 26.068)	**0.028** ^**^
Routine cleaning of milk containers.	Cleaning before putting in milk	6 (31.6)	13 (68.4)	3.69 (0.790, 17.249)	**0.097** ^*^	4.40 (0.581, 33.335)	0.151p
	Cleaning after delivery of milk	1 (25)	3 (75)	2.67 (0.206, 24.555)	0.453	2.04 (0.88, 37.422)	0.658
	Before putting in milk and after delivery of milk	3 (11.1)	24 (88.9)	1		1	
Cooling of milk	Yes	2 (10.5)	17 (89.5)	1		1	
	No	8 (25.8)	23 (74.2)	2.96 (0.556, 15.729)	**0.204** ^*^	1.7 (0.84, 11.119)	0.980
Type of containers used to collect milk	Wide necked‐plastic vessels	2 (10)	18 (90)	1		1	
	Narrow necked plastic vessels	7 (23.3)	23 (76.7)	2.74 (0.506, 14.818)	**0.242** ^*^	1.24 (0.017, 3.407)	0.292
	Traditional container	2 (20)	8 (80)	2.25 (0.267, 18.925)	0.455	1.61 (0.059, 6.185)	0.672
Main source of water	Tap water	31 (11.1)	249 (88.9)	1		1	
	River **/**Open well	18 (25.7)	52 (74.3)	2.79 (1.447, 5.343)	**0.002** ^*^	1.95 (0.705, 5.363)	0.199
Habit of milk consumption	Boiled milk	5 (6.6)	71 (93.4)	1		1	
	Raw milk	44 (16.1)	230 (83.9)	2.72 (1.038, 7.112)	0.042*	1.38 (0.190‐1.633)	0.195
Knowing any GIT disturbance	Yes	11 (8.2)	123 (91.8)	1		1	
	No	38 (17.6)	178 (82.4)	2.39 (1.174, 4.852)	**0.016** ^*^	2.12 (0.563, 7.992)	0.267

Abbreviations: AOR = adjusted odd ratio, CI = confidence interval, COR = crude odd ratio, 1 = reference group, GIT = gastro intestinal tract.

** Variables significant at *p* < 0.05

* Candidate variables for multivariate analysis at *p* < 0.25.

### 3.7 Antibiotic Susceptibility Patterns of *S. aureus*

As illustrated in Table 6, antimicrobial susceptibility of *S. aureus* showed high susceptibility to cefoxitin (95.9%), oxacillin (95.9%), and erythromycin (93.9%), whereas a high level of resistance was recorded against tetracycline (81.6%), penicillin G (81.6%), and amoxicillin (69.4%) (Table 6).

**TABLE 6:** Antibiotic susceptibility patterns of *S. aureus* isolated from raw camel milk in Babile district, Oromia region Ethiopia, May to July 2021(N= 49).

Anti‐microbial disk	Susceptibility patterns of *S. aureus*					
	S		I		R	
No	%	No	%	No	%
Gentamicin	33	67.3	2	4.1	14	28.6
Erythromycin	46	93.9	0	0	3	6.1
Ciprofloxacin	42	85.7	2	4.1	5	10.2
Penicillin G	9	18.4	0	0	40	81.6
Amoxicillin	13	26.5	2	4.1	34	69.4
Tetracycline	7	14.3	2	4.1	40	81.6
Cefoxitin	47	95.9	0	0	2	4.1
Oxacillin	47	95.9	0	0	2	4.1
Chloramphenicol	38	77.6	4	8.2	7	14.3
Trimethoprim‐sulfamethoxazole	14	28.6	7	14.3	28	57.1

Abbreviations: I = intermediate, R = resistance, S = susceptibility.

The overall prevalence of MDR patterns (resistance to at least one antimicrobial drug in three or more antimicrobial categories) of *S. aureus* isolated from raw camel milk was 48.97 % (n = 24/49). Among 49 *S. aureus* isolated from camel's raw milk, five (10.2%) isolates were resistant to only one antibiotic, eleven (22.44%) isolates were resistant to five antibiotics, and two (4.08%) isolates were resistant to eight antibiotics. However, two (4.08%) isolates were susceptible to all subjected antibiotics (Table 7).

**TABLE 7:** Multidrug resistance combination of *S. aureus* isolates from raw camel milk in Babile district, Oromia Ethiopia, May to July 2021(n=49).

Resistant to drug combination	Antimicrobial phenotypes	Number of isolates	
		Number	Percent (%)
One antibiotic	AML	2	4.08
	TET	3	6.122
Two antibiotics	PEN, TET	5	10.20
	TET, AML	2	4.08
	PEN, AML	1	2.04
	PEN, STX	2	2.08
Three antibiotics	PEN, AML, STX	1	2.04
	TET, AMP, STX	1	2.04
	PEN, TET, AML	4	8.16
Four antibiotics	PEN, TET, AML, STX	6	12.24
	PEN, GEN, TET, STX	2	4.08
	PEN, TET, CHL, STX	1	2.04
Five antibiotics	PEN, GEN, TET, AML, STX	7	14.28
	CHL, PEN, TET, AML, STX	2	4.08
	CIP, PEN, GEN, TET, AML	1	2.04
	CHL, CIP, PEN, TET, AML	1	2.04
Six antibiotics	CHL, PEN, GEN, TET, AML, STX	2	4.08
	ERY, CIP, PEN, GEN, AML, STX	1	2.04
	ERY, CIP, PEN, TET, AML, STX	1	2.04
Eight antibiotics	CHL, CIP, PEN, TET, AML, CFO, OX, STX	1	2.08
	ERY, PEN, GEN, TET, AML, CFO, OX, STX	1	2.08
None	Resistance to none (susceptible to all antibiotics)	2	4.08
Total		49	100

Abbreviations: AML = amoxicillin, CFO = cefoxitin, CHL = chloramphenicol, CIP = ciprofloxacin, ERY = erythromycin, GEN = gentamicin, OX = oxacillin, PEN = penicillin G, STX = trimethoprim‐sulfamethoxazole, TET = tetracycline.

## 4 Discussion

Camel milk is a major food source in arid and sub‐arid environments, fulfilling a variety of qualitative and quantitative dietary requirements. Pastoralists in Ethiopia's eastern lowlands rely heavily on camels due to their outstanding ability to thrive in arid and semi‐arid settings with limited forage and water resources (Bekele and Molla 2001).

In the current study, the prevalence of *S. aureus* was 14% (95% CI: 10%–18%). The prevalence within the sources of sampling points varied from 10.8 %, 12.6%, 18.3% and 20% isolated from the udder, milking container, primary milk collection centre and vendor's milk container, respectively. This finding was in line with a study conducted in Ethiopia, in the Somali region 11.27% (Teshome et al., 2016); in the Jigjiga district, at 11.45% (Serda et al., 2018); in Iraq, in Al‐Qadisyia Province 13.4% (Abdulkadhim, 2012); and in Somalia, among Borana pastoralist 10.8% (Kebede et al., 2019). However, the present finding was higher than the study done in Eastern Libya (2.7%) (Abdurabbah et al. 2018); around Jigjiga town (4.2%) (Husein et al., 2013); and the study conducted in Iran (2.72%) (Rahi et al., 2020). In contrast, the present study was lower than the study done in Saudi Arabia, 20% (Aljahani et al., 2020); in Matrouh, Egypt, 46% (Ayoub et al., 2020); in Punjab, Pakistan, 88.5% (Aqib et al., 2017); in Southern Algeria, 35.61% (Saidi et al., 2021); in Algeria, 21% (Chaalal et al., 2016); in Kafr Elsheikh, Egypt, 22.6% (Tawfik et al., 2019); and in Dubti town, Afar region, 54% (Wasie et al., 2015). This discrepancy might be due to variability in geographical area, climate condition, system of rearing, awareness of hygiene of milking practice, and variations in the study methods and materials employed by the investigators.

In the present study, most of the milking personnel (94.2%) did not wash the udder before milking, and 78.6 % of the milking personnel did not use a separate towel to dry the udder after washing. This result was in agreement with a study done around Jigjiga city of the Somali Region that reported about 92% of respondents did not use udder washing before milking (Husein et al. 2013). However, the finding disagrees with a study conducted in southern Ethiopia (Gebremedhin et al. 2020) which reported 93.3% of milking personnel were washed udder before milking. The discrepancy might be due to the difference in awareness of milking personnel on milking practice in the study area.

Furthermore, 80% of respondents used tap water as their main source of water used for cleaning, and 20% used river/open well water as their main source of water for cleaning, but the difference was not statistically significant. The result was nearly in line with the study done in southern Ethiopia (Gebremedhin et al., 2020) that reported 97.5% and 11.8 % of respondents used tap water and well water, respectively.

The current study revealed that the prevalence of *S. aureus* increases as parity increases. Raw milk from lactating camels who gave many births was 3.52 times more likely to be contaminated with *S. aureus* when compared with raw milk from those who gave few and moderate births [AOR = 3.52; 95% CI: (1.104, 13.057); *p* ≤ 0.021]. The result was in agreement with a study done in Pakistan (Aqib et al., 2017) and a study done in Mukaturi and Sululta town of Oromia Region (Sema et al., 2019). This could be because as the parity increases, there is a high degree of contamination of the udder and milk through the milking process. Besides, a large amount of milk produced and the pressure on the teat canal forces the canals to be opened widely, allowing the entrance of microbes (Sema et al. 2019).

In the present study, the prevalence of *S. aureus* in raw camel milk was significantly associated with milking personnel who did not wash hands before milking. Raw milk from milking personnel who did not wash their hands before milking was 3.94 times more likely to be contaminated with *S. aureus* when compared with milk from those washed hands before milking [AOR = 3.94: 95% CI: (1.599, 9.716); *p* = 0.003]. This result was in line with a study conducted in Kenyan pastoral herds (Kashongwe et al. 2017). However, it disagrees with the study conducted in Nasarawa, Nigeria (Yakubu et al., 2020), which reported hand washing before milking was not statistically significant (*p* = 0.800). Sanitary milking habits are important to avoid the spreading of bacteria or their proliferation. The predominant source of the infection is the udder of infected camels, transmitted through milker's hands, utensils, towels and the environment (floor) in which the camels are kept (Elemo et al. 2017).

Prevalence of *S. aureus* in raw camel milk was significantly associated with drainage condition of milking place. Milk from poor drainage conditions of milking place was 4.62 times more likely to be contaminated with *S. aureus* compared to milk from good drainage condition of milking place [AOR = 4.62, 95% CI: (1.853, 11.557); *p* = 0.001]. The study was comparable with a previous study from Sululta Town, Oromia Region, Ethiopia (Sema et al. 2019), which reported the drainage condition of the milking area was significantly (x^2^ = 4.448, *p* = 0.035) associated with the prevalence of *S. aureus* in raw milk. Milking area should minimize the risk of contamination from any source, including accumulations of dung and slurry, dust, flies, birds or other animals (Clinical and Laboratory Standards Institute (CLSI) 2006).

The current study showed that the type of containers used for selling milk was associated with the prevalence of *S. aureus* in raw camel milk. Raw milk from vendors who utilise plastic water bottles as containers for selling milk were 8.4 times more likely to be contaminated when compared with raw milk from those who use wide‐necked aluminium container, wide‐ necked plastic containers and narrow‐necked plastic containers [AOR = 8.40, 95% CI: (1.258, 26.068) *p* = 0.028]. The use of plastic containers is not advisable, as it is sensitive to heat, and their surface was easily scratched by common cleaning systems. As a result, the surface is nearly impossible to clean with the common cleaning systems and provides hiding places for bacteria during sanitisation, which allows the multiplication of bacteria on milk contact surfaces (Pandey and Voskuil 2011).

In this current study, the antimicrobial susceptibility patterns of *S. aureus* against ten antimicrobial agents showed that all isolates were found to be 95.9% susceptible to cefoxitin and oxacillin, while 69.4% were resistant to amoxicillin. This result was nearly in agreement with a study done in Bangladesh (Jahan et al., 2015) and a study done by Rana et al. (2019) who reported 91.2% of isolated *S. aureus* were susceptible to oxacillin. In addition, the current study indicated 93.9% (46/49) of isolated *S. aureus* were susceptible to erythromycin, while 6.1% (3/49) were resistant to erythromycin. These results were comparable with the study done in China (Wei et al. 2018), in Nigeria (Yakubu et al. 2020) and in Kombolcha, Northern Ethiopia (Mesfin 2015). This might be because these antibiotics are not frequently used in the study area in veterinary services and perhaps in human medicine.

However, most of the isolated *S. aureus* were resistant to penicillin G (81.6%; n = 40 /49) and tetracycline (81.6%; n = 40 /49), which was comparable to the study conducted around Asella town, Arsi zone (Elemo et al. 2017), in Kazerun, Iran (100% resistance for both penicillin and tetracycline) (Rahi et al. 2020), and in Jigjiga city (Melese et al. 2016). Furthermore, the result was in line with the study done in Central Ethiopia (Emeru et al. 2019). The possible reason of the presence of high antibiotic resistance of *S.aureus* to tetracycline, penicillin G and amoxicillin is the indiscriminate and repeated use of these antibiotics in animal and human health. Furthermore, tetracycline and penicillin are the most commonly used antimicrobials in the treatment of infections in the livestock sector in Ethiopia (Gemeda et al. 2020), and many strains of *S. aureus* are now resistant to penicillin because they develop an enzyme known as beta‐lactamase or penicillinase (Van et al. 2020).

The present study showed that 85.7% (42/49) of *S. aureus* isolates were susceptible to ciprofloxacin, while two (4.1%) and five (10.2%) were intermediate and resistant, respectively. The current finding was nearly in agreement with a study conducted in Pakistan (100% susceptible) (Aqib et al. 2017), in part of Kaduna State, Nigeria (100% susceptible) (Okpo et al. 2018), and Algeria (100% susceptible) (Yakubu et al. 2020). In addition, the current study revealed that 77.6% of the isolated *S. aureus* were susceptible to chloramphenicol, while 14.3% were resistant. The finding was nearly in agreement with a study conducted in Al Jabal Al Akhdar of Eastern Libya (Abdurabbah et al. 2018) and a study in Jigjiga City of Somali Region (Melese et al. 2016). Moreover, the present study illustrated 67.3% of isolated *S. aureus* were susceptible to gentamicin and 28.6% were resistant. The finding was in nearly agreement with a study conducted in China (Jingsha et al. 2019).

The overall prevalence of the MDR rate of *S. aureus* isolated in this study was 48.97 % (n = 24/49). The most frequent MDR isolates were those exhibiting resistance to penicillin G, ampicillin, gentamicin, tetracycline and trimethoprim‐sulfamethoxazole at a frequency of 14.28 %. The present finding of the MDR rate of *S. aureus* isolated in this study was in line with the previous study conducted in Jigjiga City of the Somali Regional State (55.2 %) (Melese et al., 2016) and a study done in Asella town, Arsi zone (52.05%) (Elemo et al. 2017).

The resistance pattern of *S. aureus* against broad‐spectrum antibiotics illustrates an alarming situation, which needs special attention. The increasing number of MDR might be due to extensive misuse of antibiotic treatment in veterinary and the use of antibiotics for the preservation of milk. Moreover, tetracycline was used as a growth promoter in food animal production (Normanno 2005).

## 5 Conclusion and Recommendation

The study revealed that the overall prevalence of *S. aureus* isolated from raw camel milk in the Babile district was high, and the isolated *S. aureus* showed a 48.97% MDR rate. The isolated *S. aureus* from raw camel milk was more resistant to tetracycline and penicillin G, whereas cefoxitin, oxacillin and erythromycin were the most effective antimicrobial agents against *S. aureus*. Milk handling practice and parameters, including drainage condition of the milking place, not hand washing before milking, type of containers used for selling milk and parity of lactating camels, were statistically significant predictors of *S. aureus* prevalence in raw camel milk. Most of the respondents consumed raw milk without any heat treatment, and the milk was not cooled both after milking and before delivery to the market due to lack of chilling facilities. Thus indicating the possibilities for *S. aureus* milk contamination and intoxication that was a risk for consumers. The result indicates that the raw camel milk samples were produced and handled under poor hygienic conditions, posing a significant health risk to consumers. Consumption of raw camel milk should get concerns from concerned bodies. Based on this finding, we recommend the immediate development and implementation of effective, practical, and sustainable interventions to improve camel milk hygiene and safety in the study area, as well as to reduce staphylococcal food poisoning in the area.

## Author Contributions

All authors made a significant contribution to the work reported, either in the conception, or the design of the study, conducting the actual study, collection of data, analysis and interpretation of data, or drafting the manuscript. All authors were critically revising the manuscript for important intellectual content and reviewed the final version to be published. We all have decided to which journal the article will be submitted. We all agree to be responsible and accountable for any contents of the article.

## Conflicts of Interest

The authors declare no conflicts of interest.

### Peer Review

The peer review history for this article is available at https://www.webofscience.com/api/gateway/wos/peer‐review/10.1002/vms3.70438.

## Supporting information




**Supporting Fig. 1**: Mannitol salt agar media with growth of *S.aureus* mannitol fermenter colony.
**Supporting Fig. 2**: A blood agar media with β‐hemolysis colony of *S.aureus*.
**Supporting Fig. 3**: Antibiotic susceptibility test with different antibiotic.

## Data Availability

Due to the privacy policy, the datasets are not publicly available. On reasonable request, the corresponding author will provide the data that support the findings of this study.

## References

[vms370438-bib-0001] Abdulkadhim, M. 2012. “Prevalence of Methicillin Resistance *Staphylococcus aureus* in Cattle and She‐camels Milk at Al‐Qadisyia Province.” Al‐Anbar Journal of Veterinary Sciences 5, no. 2: 63–67.

[vms370438-bib-0002] Abdurabbah, E. M. , N. M. Eissa , S. M. Hussieny , and Y. I. Mahmoud . 2018. “Fares NH. Isolation of Coagulase Positive *Staphylococci* From She‐Camel Milk at Eastern Libya and Their Drug Susceptibility Patterns.” Australian Journal of Basic and Applied Sciences 12, no. 8: 118–123.

[vms370438-bib-0003] Aljahani, A. H. , K. M. Alarjani , Z. K. Hassan , et al. 2020. “Molecular Detection of Methicillin Heat‐resistant *Staphylococcus aureus* Strains in Pasteurized Camel Milk in Saudi Arabia.” Bioscience Reports 40, no. 4. 10.1042/BSR20193470 PMC7167254 32202302

[vms370438-bib-0004] Amentie, T. , M. Eshetu , Y. Mekasha , and A. Kebede . 2016. “Milk Postharvest Handling Practices Across the Supply Chain in Eastern Ethiopia.” Journal of Advanced Veterinary and Animal Research 3, no. 2: 112–126.

[vms370438-bib-0005] Ana, G. A. , G. V. Tomás , and B.‐V. Jorge , et al. 2020. “ *Staphylococcus aureus* Exotoxins and Their Detection in the Dairy Industry and Mastitis.” Toxins MDPI 12: 537. 10.3390/toxins12090537 PMC7551672 32825515

[vms370438-bib-0006] Aqib, A. I. , M. Ijaz , A. Z. Durrani , et al. 2017. “Prevalence and Antibiogram of *Staphylococcus aureus*, a Camel Mastitogen From Pakistan.” Pakistan Journal of Zoology 49, no. 3: 861–867.

[vms370438-bib-0007] Aqib, A. I. , M. Ijaz , S. H. Farooqi , and A. Raza . 2018. Dairy *Staphylococcus aureus*: Epidemiology, Drug Susceptibilities, Drug Modulation, and Preventive Measures. Staphylococcus aureus: IntechOpen.

[vms370438-bib-0008] Aqib, A. I. , M. Ijaz , R. Hussain , et al. 2017. “Identification of Coagulase Gene in *Staphylococcus aureus* Isolates Recovered From Subclinical Mastitis in Camels.” Pakistan Veterinary Journal 37, no. 2: 160–164.

[vms370438-bib-0009] Aqib, A. I. , S. Nighat , A. Rais , et al. 2019. “Drug Susceptibility Profile of *Staphylococcus aureus* Isolated From Mastitic Milk of Goats and Risk Factors Associated With Goat Mastitis in Pakistan.” Pakistan Journal of Zoology 51, no. 1: 307–315.

[vms370438-bib-0010] Arumugam, G. , H. Periasamy , and P.‐S. Maneesh . Staphylococcus aureus: Overview of Bacteriology, Clinical Diseases, Epidemiology, Antibiotic Resistance and Therapeutic Approach. 2017. 1–27.

[vms370438-bib-0011] Ayoub, E. S. , A. A. Amer , H. G. Keshta , and E. K. Sedeek . 2020. “Microbial Assessment of Raw Dromedary Camel Milk in Matrouh Governorate, Egypt.” Alexandria Journal for Veterinary Sciences. 66, no. 2: 40–47.

[vms370438-bib-0012] BDLDHA (Babile District Livestock Development and Health Agency) . Annual Report on Livestock Production Potential at Babile District Ethiopia. 2018. 113.

[vms370438-bib-0013] Bekele, T. , and B. Molla . 2001. “Mastitis in Lactating Camels (Camelus dromedarius) in Afar Region, North‐Eastern Ethiopia.” Berliner Und Munchener Tierarztliche Wochenschrift 114, no. 5–6: 169–172. 11413707

[vms370438-bib-0014] Betelihem, T. , and T. Shimels . 2017. “Bacteriological Milk Quality: Possible Hygienic Factors and the Role of *Staphylococcus aureus* in Raw Bovine Milk in and Around Gondar.” Ethiopia. International Journal of Food Contamination. 4, no. 1: 1–9.

[vms370438-bib-0015] Carruth, L. , A. A. Roess , Y. Terefe , F. M. Hosh , and M. Salman . 2017. “Antimicrobial Resistance and Food Safety in Africa.” The Lancet Infectious Diseases 17: 575–576. 28555570 10.1016/S1473-3099(17)30273-6

[vms370438-bib-0016] Chaalal, W. , H. Aggad , K. Zidane , N. Saidi , and M. Kihal . 2016. “Antimicrobial Susceptibility Profiling of *Staphylococcus aureus* Isolates From Milk.” Microbiology Research Journal International 13: 1–7.

[vms370438-bib-0017] Clinical and Laboratory Standards Institute (CLSI) . 2017. “Performance Standards for Antimicrobial Susceptibility testing.” 27th ed. CLSI Supplement M100S, Wayne, PA: CLSI;. 37: 1–249.

[vms370438-bib-0018] CSA . 2014. Federal Democratic Republic of Ethiopia, Central Statistical Agency, Addis Ababa, Ethiopia. Population Projection of Ethiopia for the Year 2014, 4–38.

[vms370438-bib-0019] Dairy safe . *Guidelines for the Safe Manufacture of Dairy Products*: Government of South Australia 2019, 98.

[vms370438-bib-0020] Danai, E. , S. Jenny , S. Markus , and J. Sophia . 2020. “Staphylococcal Enterotoxin C—An Update on SEC Variants, Their Structure and Properties, and Their Role in Foodborne Intoxications.” Journal of Toxins 1–17.. 32927913 10.3390/toxins12090584 PMC7551944

[vms370438-bib-0021] Dhanashekar, R. , S. Akkinepalli , and A. Nellutla . 2012. “Milk‐borne Infections. An Analysis of Their Potential Effect on the Milk Industry.” Germs 2, no. 3: 101.. 24432270 10.11599/germs.2012.1020 PMC3882853

[vms370438-bib-0023] Edalati, E. , B. Saneei , M. Alizadeh , et al. 2019. “Isolation of Probiotic Bacteria From Raw Camel's Milk and Their Antagonistic Effects on Two Bacteria Causing Food Poisoning.” New Microbe and New Infect 27: 64–68. 10.1016/j.nmni.2018.11.008 PMC6317326 30622712

[vms370438-bib-0024] Elemo, K. , T. Tessema , A. Shiferaw , and M. Fato . 2017. “Prevalence, Risk Factors and Multidrug Resistance Profile of *Staphylococcus aureus* Isolated From Bovine Mastitis in Selected Dairy Farms in and Around Asella town, Arsi Zone, South Eastern Ethiopia.” African Journal of Microbiology Research 11: 1632–1642.

[vms370438-bib-0025] Emeru, B. A. , Y. E. Messele , D. T. Tegegne , et al. 2019. “Characterization of Antimicrobial Resistance in *Staphylococcus aureus* Isolated From Bovine Mastitis in Central Ethiopia.” Journal of Veterinary Medicine and Animal Health 11, no. 4: 81–87.

[vms370438-bib-0026] Emmanuella, O. M. , H. N. R. Adriano , S. M. Cláudia , A. U. Stela , P. N. Luciano , and M. L. J. Dorgival . 2019. “Enterotoxin‐encoding Genes in *Staphylococcus aureus* From Buffalo Milk.” Veterinária Brasileira 39, no. 8: 587–591.

[vms370438-bib-0027] Enquebaher, K. T. 2016. Staphylococcus aureus From Milk and Milk Products in Ethiopia: Prevalence, Enterotoxigenic Potential, Antibiotic Resistance and Spa Types. Oslo, Norway: Norwegian University of Life Sciences.

[vms370438-bib-0028] Ewa, Z. , G. Jan , and T. Magdalena . 2016. “Food‐borne Pathogens and Contaminants in Raw Milk–A Review.” Annals of Animal Science 16, no. 3: 623–639.

[vms370438-bib-0029] Fakhri, H. , D. Shahrzad , P. Angineh , and Z. Habib . 2019. “The Frequency of *Staphylococcus aureus* Classical Enterotoxin Genes in Raw Milk Samples in Zanjan, Iran.” Journal of Human, Environment, and Health Promotion 5, no. 1: 32–35.

[vms370438-bib-0030] Food Hygiene Regulations . A Practical Guide for Milk Producers to the Food Hygiene Regulations. 2006. 6.

[vms370438-bib-0031] Garcia, S. N. , B. I. Osburn , J.‐R. MT . 2020. “One Health for Food Safety, Food Security, and Sustainable Food Production.” Frontiers in Sustainable Food Systems 4: 1.

[vms370438-bib-0032] Gebremedhin, S. G. , S. E. Mequnnent , and A. A. Gichamo . 2020. “Assessment of Knowledge, Attitudes and Practices of People About Milk Quality and Common Zoonotic Diseases in Small Holder Dairy Production Chain in Selected Sites of Southern Ethiopia.” International Journal of Advanced Research in Biological Sciences 7, no. 8: 25–36.

[vms370438-bib-0033] Gemeda, B. A. , K. Amenu , U. Magnusson , et al. 2020. “Antimicrobial Use in Extensive Smallholder Livestock Farming Systems in Ethiopia: Knowledge, Attitudes, and Practices of Livestock Keepers.” Frontiers in Veterinary Science 7: 55. 32175334 10.3389/fvets.2020.00055 PMC7055293

[vms370438-bib-0034] Gemeda, B. A. , K. Amenu , U. Magnusson , et al. 2020. “Antimicrobial Use in Extensive Smallholder Livestock Farming Systems in Ethiopia: Knowledge, Attitudes, and Practices of Livestock Keepers.” Frontiers in Veterinary Science 7: 55. 32175334 10.3389/fvets.2020.00055 PMC7055293

[vms370438-bib-0035] Guliye, A. , I. Noor , B. Bebe , and I. Kosgey . 2007. “Role of Camels (Camelus dromedarius) in the Traditional Lifestyle of Somali Pastoralists in northern Kenya.” Outlook on Agriculture 36, no. 1: 29–34.

[vms370438-bib-0036] Husein, A. , B. Haftu , A. Hunde , and A. Tesfaye . 2013. “Prevalence of Camel (Camelus dromedaries) Mastitis in Jijiga Town.” Ethiopia. African Journal of Agricultural Research. 8, no. 24: 3113–3120.

[vms370438-bib-0037] Jahan, M. , M. Rahman , M. S. Parvej , et al. 2015. “Isolation and Characterization of *Staphylococcus aureus* From Raw Cow Milk in Bangladesh.” Journal of Advanced Veterinary and Animal Research 2, no. 1: 49–55.

[vms370438-bib-0038] Jingsha, D. , W. Shi , H. Jiahui , et al. 2019. “Prevalence and Characterization of *Staphylococcus aureus* Isolated From Pasteurized Milk in China.” Frontiers in Microbiology 10, no. 641: 1–10.. 31001225 10.3389/fmicb.2019.00641 PMC6454862

[vms370438-bib-0039] Kashongwe, O. B. , B. O. Bebe , J. W. Matofari , and C. G. Huelsebusch . 2017. “Associations Between Milking Practices, Somatic Cell Counts and Milk Postharvest Losses in Smallholder Dairy and Pastoral Camel Herds in Kenya.” International Journal of Veterinary Science and Medicine 5, no. 1: 57–64. 30255050 10.1016/j.ijvsm.2017.01.001 PMC6137855

[vms370438-bib-0040] Kebede, A. , G. Delia , N. Shemsu , and B. Wieland . 2019. “Bacteriological Quality and Safety of Ready‐to‐Consume Milk and Naturally Fermented Milk in Borana Pastoral Area, Southern Ethiopia.” Tropical Animal Health and Production 51, no. 7: 2079–2084. 30919322 10.1007/s11250-019-01872-8

[vms370438-bib-0041] Melese, A. R. , W. B. Tesfaye , and N. A. Ayalew . 2016. “Bacterial Contaminations of Raw Cow's Milk Consumed at Jigjiga City of Somali Regional State, Eastern Ethiopia.” International Journal of Food Contamination 3, no. 4: 1–9.

[vms370438-bib-0042] Mesfin, N. 2015. Isolation, Identification and Drug Resistance Patterns of Methicilin Resistant Staphylococcus aureus From Mastitic Cow's Milk From Selected Dairy Farms in and Around Kombolcha. Bishoftu, Ethiopia: Addis Ababa University.

[vms370438-bib-0043] Normanno, G. 2005. “Coagulase‐positive staphylococci and *Staphylococcus aureus* in Food Products Marketed in Italy.” Food Microbiology International Journal 98: 73–79. 10.1016/j.ijfoodmicro.2004.05.008 15617802

[vms370438-bib-0044] Okpo, N. , I. Abdullahi , C. Whong , and J. Ameh . 2018. “Occurrence and Antibiogram of *Staphylococcus aureus* in Some Dairy Products Sold in Parts of Kaduna State, Nigeria.” Journal of Microbiology Research 3, no. 1: 56–60.

[vms370438-bib-0045] Pandey, G. , and G. Voskuil . 2011. “Manual on Milk Safety, Quality and Hygiene.” Golden Valley Agricultural Research Trust, Zambia 1–52.

[vms370438-bib-0046] Quinn, P. , M. Carter , B. Markey , and G. Carter . 2004. Mastitis in Clinical Veterinary Microbiology. Mosby International Limited London. 327–344.

[vms370438-bib-0047] Rahi, A. , H. Kazemeini , S. Jafariaskari , A. Seif , S. Hosseini , and F. S. Dehkordi . 2020. “Genotypic and Phenotypic‐Based Assessment of Antibiotic Resistance and Profile of Staphylococcal Cassette Chromosome Mec in the Methicillin‐Resistant *Staphylococcus aureus* Recovered From Raw Milk.” Infection and Drug Resistance 13: 273. 32099419 10.2147/IDR.S229499 PMC6996610

[vms370438-bib-0048] Rana, T. A. , T. Dalia , A. TM , and I. MS . 2019. “Detection of Staphylococci in Milk Samples in Retails in Kafr Elsheikh Governorate, Egypt.” European Journal Of Pharmaceuticaland Medical Research 6, no. 4: 92–97.

[vms370438-bib-0049] Remaz, M. , N. Juma , and B. Elhag . 2017. “Characterization of *Staphylococcus* Species and Detection of Methicillin Resistant *Staphylococcus aureus* in Camel Milk at Khartoum North, Sudan.” International Journal of Science and Research (IJSR) 6, no. 6: 1067–1072.

[vms370438-bib-0050] Saidi, R. , N. Mimoune , M. H. Benaissa , et al. 2021. “Camel Mastitis in Southern Algeria.” Veterinarska Stanica 52, no. 3: 315–322.

[vms370438-bib-0051] Sema, R. , M. Shimelis , and A. Ashebr . 2019. “Milk Safety Assessment, Isolation, and Antimicrobial Susceptibility Profile of *Staphylococcus aureus* in Selected Dairy Farms of Mukaturi and Sululta Town, Oromia Region, Ethiopia.” Hindawi Veterinary Medicine International 1: 11. 10.1155/2019/3063185 PMC6699244 31467658

[vms370438-bib-0052] Serda, B. , A. Bekele , and D. Abebe . 2018. “Prevalence and Contamination Level of *Staphylococcus aureus* in Raw Camel Milk and Associated Risk Factors in Jigjiga District, Eastern Ethiopia.” Journal of Veterinary Science &Technology 9, no. 1: 1–5.

[vms370438-bib-0053] Tawfik, R. , D. Talat , T. Azer , and M. Ibrahim . 2019. “Detection of *Staphylococci* in Milk Samples in Retails in Kafr Elsheikh Governorate, Egypt.” European Journal of Pharmaceutical and Medical Research 6, no. 4: 92–97.

[vms370438-bib-0054] Teshome, B. , G. Tefera , B. Belete , and A. Mekuria . 2016. “Prevalence and Antimicrobial Susceptibility Pattern of *Staphylococcus aureus* From Raw Camel and Goat Milk From Somali region of Ethiopia.” African Journal of Microbiology Research 10, no. 28: 1066–1071.

[vms370438-bib-0055] Van, T. T. H. , Z. Yidana , P. M. Smooker , and P. J. Coloe . 2020. “Antibiotic Use in Food Animals Worldwide, With a Focus on Africa: Pluses and Minuses.” Journal of Global Antimicrobial Resistance 20: 170–177.. 31401170 10.1016/j.jgar.2019.07.031

[vms370438-bib-0056] Venugopal, N. , S. Mitra , R. Tewari , et al. 2019. “Molecular Detection and Typing of Methicillin‐resistant *Staphylococcus aureus* and Methicillin‐resistant Coagulase‐negative staphylococci Isolated From Cattle, Animal Handlers, and Their Environment From Karnataka, Southern Province of India.” Veterinary World 12, no. 11: 1760. 32009754 10.14202/vetworld.2019.1760-1768 PMC6925040

[vms370438-bib-0057] Wasie, A. , M. Pal , and F. Zeru . 2015. “A Study on Assessment of Microbial Quality of Raw Camel Milk in Dubti, Ethiopia.” The Haryana Veterinarian 54: 184–187.

[vms370438-bib-0058] Wei, W. , L. Xiaohui , and J. Tao , et al. 2018. “Prevalence and Characterization of *Staphylococcus aureus* Cultured from Raw Milk Taken From Dairy Cows With Mastitis in Beijing, China.” Frontiers in Microbiology 9: 1–6. 29988423 10.3389/fmicb.2018.01123 PMC6024008

[vms370438-bib-0059] Woldearegay, Y. H. , M. Berhanu , and A. T. Mebratu . 2015. “Study on Management Practices and Production Constraints of Camel in Raya‐Azebo District, Tigray, Northern Ethiopia.” European Journal of Biological Sciences 7, no. 1: 01–06.

[vms370438-bib-0060] Yakubu, A. , I. Abdullahi , C. Whong , and B. Olayinka . 2020. “Prevalence and Antibiotic Susceptibility Profile of *Staphylococcus aureus* From Milk and Milk Products in Nasarawa State.” Nigeria. Sokoto Journal of Veterinary Sciences. 18, no. 1: 1–12.

[vms370438-bib-0061] Yenealem, A. 2020. Isolation and Identification of Pathogenic Staphylococci and E. Coli From Raw Bovine Milk Collected From Milk Cooperative Centers in Hawassa, Southern Ethiopia.

