# Supplementary material for: Prevalence, Antimicrobial Susceptibility Pattern and Associated Factors of Staphylococcus Aureus Among Camel's Raw Milk in Babile District, Oromia, Ethiopia
Source: Vet Med Sci. 2025 Jun 1;11(4):e70438. doi: 10.1002/vms3.70438 (PMC12126996; doi:10.1002/vms3.70438)
Supplement: Supplementary file 1 — Supporting Fig. 1: Mannitol salt agar media with growth of S.aureus mannitol fermenter colony. Supporting Fig. 2: A blood agar media with β‐hemolysis colony of S.aureus. Supporting Fig. 3: Antibiotic susceptibility test with different antibiotic. [file VMS3-11-e70438-s001.docx]

Supplementary figure

Yellow color colony indicates bacteria is mannitol fermenter


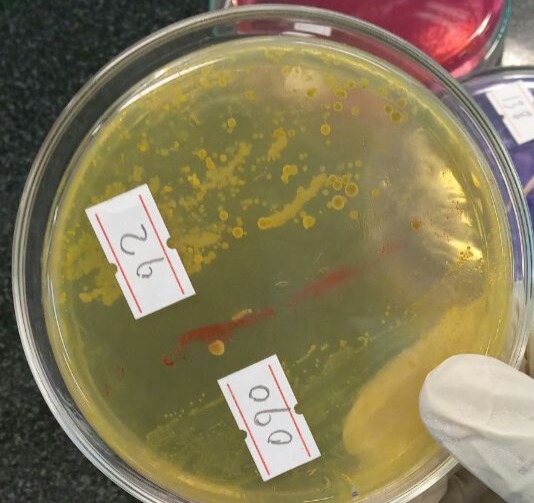


Supplementary Figure 1. Mannitol salt agar media with growth of *S.aureus* mannitol fermenter colony

Yellow arrow indicates β-hemolysis colony of *S.aureus*


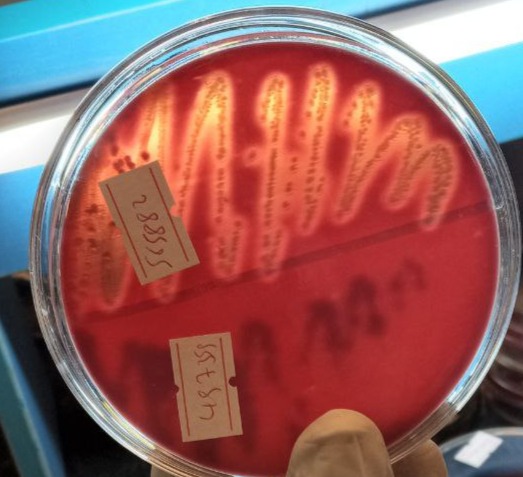


Green arrow indicates non-hemolysis colony

Supplementary Figure 2. A blood agar media with β-hemolysis colony of *S.aureus*


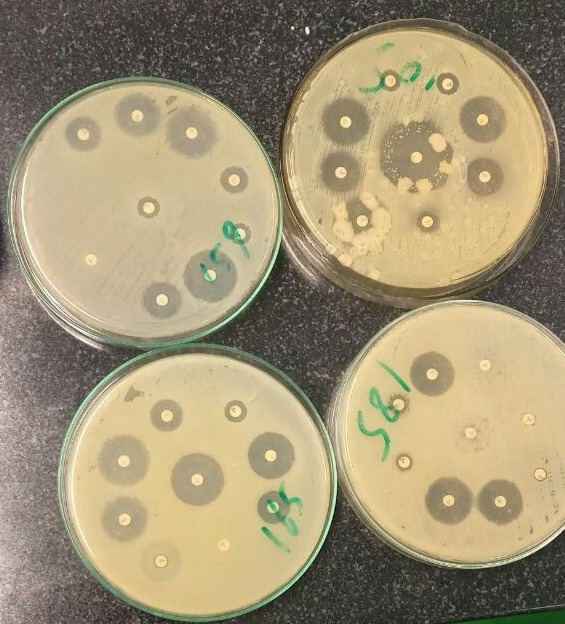


This arrow indicates antibiotic disc

This arrow indicates zone with bacterial growth

This arrow indicates zone of inhibition with susceptible organisms

This arrow indicates organism is resistant to this antibotic

Supplementary Figure 3. Antibiotic susceptibility test with different antibiotic
